# Time equals sight: Sphenoid sinus aspergilloma with vision loss

**DOI:** 10.1016/j.idcr.2022.e01440

**Published:** 2022-02-01

**Authors:** Gawahir A. Ali, Muna Al Maslamani, Mahir Petkar, Adham Ammar, Wael Goravey

**Affiliations:** a Department of Infectious Diseases, Communicable Diseases Centre, Hamad Medical Corporation, Doha, Qatar; b Department of Laboratory Medicine and Pathology, Hamad Medical Corporation, Doha, Qatar

**Keywords:** Sphenoid sinus, Aspergilloma, Visual loss, Voriconazole

## Abstract

Sphenoid sinus aspergilloma (SSA) with visual loss has rarely been reported. Timely recognition and prompt surgical intervention are crucial to avoid permanent neurological consequences.

## Introduction

**Fig. 1:**
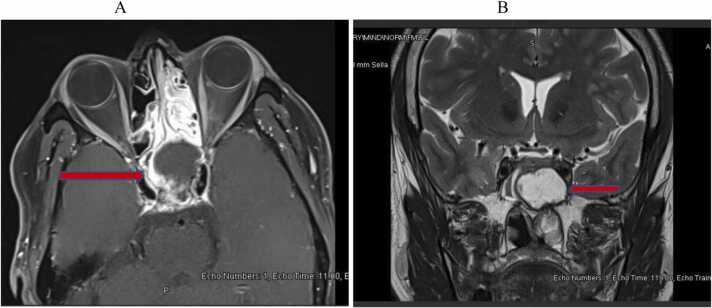
A and B: MRI brain and sinuses revealed a left sphenoid sinus mass (Arrowed) compressing the posterior wall of the clivus, greater wing of the sphenoid, and the optic nerve canal with optic nerve inflammation.

**Fig. 2:**
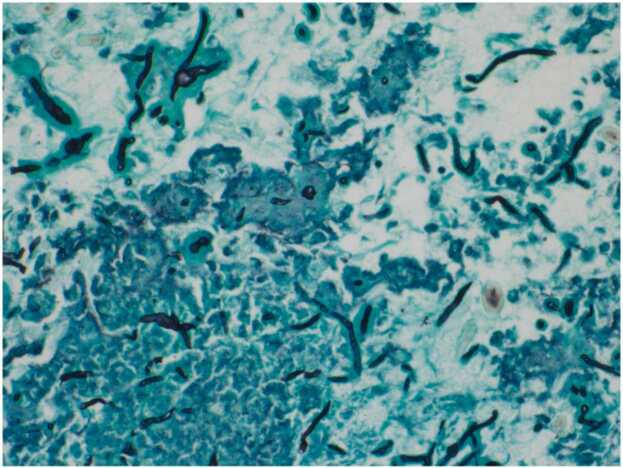
Grocott stain of the left sphenoid sinus mass highlighting abundant aspergillus spp.

A 51-year-old male was assessed for sudden, non-traumatic, painless vision loss in the left eye of 4 h duration preceded by retro-orbital headache for 2 days. He had no significant past medical conditions. Examination revealed decreased visual acuity to light perception in the left eye and no other focal neurological deficits. Urgent CT brain imaging followed by MRI revealed a left sphenoid sinus mass with dehiscence of the posterior wall of the clivus and the greater wing of the sphenoid compressing the optic nerve canal suggesting aspergilloma or tumour (Figs. 1A and 1B). He underwent urgent decompression and removal of the mass through left functional endoscopic sinus surgery (FESS). Dehiscence in the sphenoid wall, optic and carotid canals were observed. Histopathological sections revealed sinonasal mucosa with chronic inflammation and fungal elements consistent with *Aspergillus* spp (Fig. 2). On post-surgery day 2, the vision started to recover until he fully regained his visual acuity by week 4. However, considering the extensive nature of bony dehiscence and inflamed optic nerve, voriconazole was added for 3 months. Follow-up MRI after 3 months, showed improvement in optic nerve inflammation with postoperative changes. He had no recurrence 18 months into follow-up.

Aspergilloma is rarely affecting the sphenoid sinuses and can remain indolent; thus, the diagnosis is often delayed until complications occur [1]. Although noninvasive, Sphenoid sinus aspergilloma (SSA) can lead to devastating consequences due to the vicinity to crucial structures of the skull base [1]. Therefore, optic nerve compression causing vision loss is not exceptional [2]. Females and those over 50 years old are predominantly affected. *Aspergillus fumigatus* is a commonly isolated species, while headache is the main symptom [3]. Vision loss with SSA has poor visual prognosis despite surgical intervention [4]. CT is frequently used, but MRI plays an important role in delineating the involvement of adjunct structures [1]. Histopathological diagnosis is critical and FESS with the removal of the fungus ball is the current treatment modality, while antifungals are controversial [3].

## Ethical approval

Ethics approval and permission was obtained to publish the case reports from the institutional review board which is in line with international standards.

## Funding

No funding was received towards the publication.

## CRediT authorship contribution statement

**GA:** Clinical management, data acquisition and manuscript writing. **MA** supervised all the aspects and contributed to final manuscript editing. **MP and AA:** Contributed to data acquisition and histopathology reports. **WG:** Clinical management, contribute to data acquisition, manuscript preparation and final proof reading.

## Consent

Written informed consent was obtained from the patient to publish this report in accordance with the journal`s patient consent policy.

## Conflict of interest

The authors declare that they have no competing interests.

## Data Availability

The authors confirm that the datasets supporting the findings of this case are available from the corresponding author upon request.
